# A Window of Opportunity for Cognitive Training in Adolescence

**DOI:** 10.1177/0956797616671327

**Published:** 2016-11-04

**Authors:** Lisa J. Knoll, Delia Fuhrmann, Ashok L. Sakhardande, Fabian Stamp, Maarten Speekenbrink, Sarah-Jayne Blakemore

**Affiliations:** 1 Institute of Cognitive Neuroscience; 2 Department of Experimental Psychology, University College London

**Keywords:** adolescence, sensitive periods, cognitive development, learning, plasticity, cognitive training, education

## Abstract

In the current study, we investigated windows for enhanced learning of cognitive skills during adolescence. Six hundred thirty-three participants (11–33 years old) were divided into four age groups, and each participant was randomly allocated to one of three training groups. Each training group completed up to 20 days of online training in numerosity discrimination (i.e., discriminating small from large numbers of objects), relational reasoning (i.e., detecting abstract relationships between groups of items), or face perception (i.e., identifying differences in faces). Training yielded some improvement in performance on the numerosity-discrimination task, but only in older adolescents or adults. In contrast, training in relational reasoning improved performance on that task in all age groups, but training benefits were greater for people in late adolescence and adulthood than for people earlier in adolescence. Training did not increase performance on the face-perception task for any age group. Our findings suggest that for certain cognitive skills, training during late adolescence and adulthood yields greater improvement than training earlier in adolescence, which highlights the relevance of this late developmental stage for education.

Education policy tends to emphasize the importance of investing in early-childhood intervention. This policy is partly based on well-established economics accounts of the added value of early-childhood intervention (Heckman, 2006). However, there is a tension between the assumption that earlier is always better and the recent findings that the human brain continues to develop throughout childhood, adolescence, and into early adulthood.

Adolescence is the period of life between puberty and relative independence (Steinberg, 2010). Research has shown that several cortical regions in humans undergo protracted structural and functional development across adolescence (Cohen Kadosh, Johnson, Dick, Cohen Kadosh, & Blakemore, 2013; Giedd & Rapoport, 2010; Tamnes et al., 2010). Regions that undergo particularly substantial development include the prefrontal and parietal cortices, which are involved in a variety of higher cognitive skills relevant to mathematics education, including reasoning and numerical skills (Blakemore & Robbins, 2012; Dumontheil, 2014; Houdé, Rossi, Lubin, & Joliot, 2010). There is evidence that protracted development of these cognitive skills occurs during adolescence (Crone, Wendelken, Donohue, van Leijenhorst, & Bunge, 2006; Dumontheil, Houlton, Christoff, & Blakemore, 2010; Halberda, Ly, Wilmer, Naiman, & Germine, 2012). However, little is known about when these skills are most efficiently learned.

In the current study, we trained participants on one of three cognitive skills: numerosity discrimination, relational reasoning, and face perception. Numerosity discrimination is the ability to discriminate between small and large numerosities, and relational reasoning is the ability to detect abstract relationships between groups of items. These skills involve brain regions that undergo development in adolescence (Cohen Kadosh et al., 2013; Dehaene, Piazza, Pinel, & Cohen, 2003; Dumontheil et al., 2010), and performance in relational reasoning and numerosity discrimination improves during adolescence (Carey, Diamond, & Woods, 1980; Dumontheil, 2014; Halberda et al., 2012). Therefore, these skills might be expected to be particularly trainable during adolescence. In addition, both skills are relevant to education: They are correlated with mathematics performance (Dumontheil & Klingberg, 2012; Halberda et al., 2012), and relational reasoning is related to fluid intelligence, a significant predictor of educational outcomes (Chuderski, 2014).

A task involving face perception (i.e., identifying changes in faces and facial features) was included as the control training task. Face perception also improves during adolescence and may be susceptible to training, but it relies on cognitive processes and neural circuits different from those involved in the other two skills trained (Cohen Kadosh et al., 2013). We thus reasoned that there would be no transfer from face-perception training to performance in numerosity discrimination and relational reasoning, and there would be no transfer from training in numerosity-discrimination and relational-reasoning tasks to performance on a face-perception task.

Performance on each of the three training tasks was tested at Test Session 1 before training, between 3 and 7 weeks after training ended (at Test Session 2), and between 3 and 9 months after training ended (at Test Session 3; Fig. 1). In addition, we included two nontrained tasks in the test sessions—a working memory task (backward digit span) and a face-memory task—to determine whether transfer effects were evident and whether they differed between age groups.

**Fig. 1.**
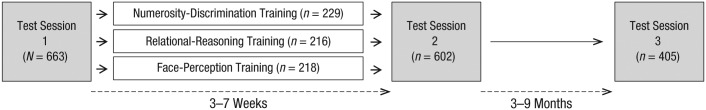
Timeline of the study and number of participants at each stage. Participants were split into three groups; one group received training in numerosity discrimination, another group received training in relational reasoning, and a third received training in face perception.

Whether training in certain cognitive skills can improve performance in nontrained skills remains under debate. Studies have reported transfer to skills that share similar cognitive processes, such as from one trained working memory task to another (Klingberg, 2010; Thorell, Lindqvist, Bergman Nutley, Bohlin, & Klingberg, 2009). A small number of studies in children and adults have found evidence for transfer to skills that are less closely related. For instance, working memory training has been found to transfer to fluid intelligence (Bergman-Nutley & Klingberg, 2014; Jaeggi, Buschkuehl, Jonides, & Perrig, 2008; Klingberg et al., 2005), arithmetic performance (Bergman-Nutley & Klingberg, 2014), and cognitive control (Klingberg et al., 2005), and reasoning training has been found to transfer to fluid intelligence (Bergman-Nutley & Klingberg, 2014; Klingberg et al., 2005; Mackey, Hill, Stone, & Bunge, 2011). However, other studies have failed to provide evidence for such transfer to cognitive skills that are less closely related (Holmes & Gathercole, 2014; Owen et al., 2010).

The goal of the current training study was to investigate certain cognitive skills and to determine when during adolescence these skills are best trained. Studies have investigated cognitive training mainly in children and adults. In the current study, we compared training effects among participants in four age groups: 186 younger adolescents (age range = 11.27–13.38 years), 186 midadolescents (age range = 13.39–15.89 years), 186 older adolescents (age range = 15.90–18.00 years), and 105 adults (age range = 18.01–33.15 years). We investigated three central hypotheses:

- Overall training effects: Training would improve performance on the trained task only.
- Age-dependent training effects: Performance on the trained task would improve after training within some or all age groups, and the strength of improvement would differ between age groups.
- Transfer effects: Training effects might generalize to performance on a nontrained task. Specifically, training in relational reasoning might lead to improvements in performance on an untrained working memory task (Klingberg, 2010), and training in face perception might lead to improvements in performance on an untrained face-memory task (Dolzycka, Herzmann, Sommer, & Wilhelm, 2014).

## Method

### Participants

Data from 821 participants were collected over a 16-month period. Adolescents were recruited from 16 schools in and around London. Adults were recruited through the University College London participant pools (which are databases that include individuals who are not students and have not previously studied at University College London) and through posters in central London, near the university. School-age participants were tested during lessons, and data were collected from all students present in the classroom. Data from 123 students were excluded because parental consent was not provided. Participants’ data were also excluded if they reported a diagnosis of developmental conditions, including attention-deficit/hyperactivity disorder, autism, dyscalculia, dyslexia, and epilepsy (*n* = 34), or if they were not present during testing at Test Session 1 (*n* = 1). The final sample at Test Session 1 included 663 participants (398 females; mean age = 16.50 years, *SD* = 4.42, age range = 11.27–33.15 years) and was divided into four age groups: younger adolescents, midadolescents, older adolescents, and adults. To create the three adolescent age groups, we sorted the 11- to 18-year-olds by age and then split them into three bins of equal size. We chose three age groups for adolescents as a compromise between the increased sensitivity that comes with increasing numbers of groups and the loss of power this engenders. Adults were tested separately from adolescents and were assigned to their own age group.

Participants were randomly assigned to one of three training groups: numerosity discrimination (*n* = 229), relational reasoning (*n* = 216), and face perception (*n* = 218) (for gender split and attrition between test sessions, see Table 1). Including a face-perception training group as well allowed us to control for nonspecific aspects of participating in a training study, such as adhering to a training schedule, online training over several days, and so forth (Klingberg, 2010). Experimenters were blind to participant training group. We tested whether training groups and age groups differed in a number of potentially confounding variables: the amount of training completed, the days between training sessions, the days between Test Sessions 1 and 2, days between Test Sessions 2 and 3, group size at testing, test sessions split over multiple days, and missing data at Test Sessions 2 and 3. There were no differences between training groups on any of these variables, but there were age-group differences on all of them (see Table S6 in the Supplemental Material available online). We therefore carried out supplemental analyses to test whether these potential confounds with age influenced our main results (see Supplementary Analyses in the Supplemental Material).

**Table 1.** Number of Participants and Gender Split for Each Age Group and Training Group at Test Sessions 1, 2, and 3 (TS1, TS2, and TS3)

	Training group										
	Numerosity discrimination			Relational reasoning			Face perception		
Age group and sample size	TS1	TS2	TS3	TS1	TS2	TS3	TS1	TS2	TS3
Younger adolescents (ages 11.27–13.38)									
Total (*n*)	62	57	37	61	56	38	63	58	43
Females (*n*)	41	39	26	32	30	26	45	42	31
Males (*n*)	21	18	11	29	26	12	18	16	12
Midadolescents (ages 13.39–15.89)									
Total (*n*)	60	57	38	63	61	46	63	59	46
Females (*n*)	30	28	21	33	33	23	27	25	22
Males (*n*)	30	29	17	30	28	23	36	34	24
Older adolescents (ages 15.90–18.00)									
Total (*n*)	71	60	42	57	49	33	58	43	25
Females (*n*)	41	37	30	33	30	21	35	26	14
Males (*n*)	30	23	12	24	19	12	23	17	11
Adults (ages 18.01–33.15)									
Total (*n*)	36	36	17	35	34	22	34	32	18
Females (*n*)	28	28	14	25	24	15	28	27	17
Males (*n*)	12	12	3	10	10	7	6	5	1

### Experimental design

Participants were tested at three test sessions (Fig. 1). They were asked to complete 20 sessions of online training between Test Sessions 1 and 2 on one of the three training tasks (numerosity discrimination, relational reasoning, or face perception). Participants were tested on five tasks at each test session: numerosity discrimination, relational reasoning, face perception, face memory, and backward digit span. The face memory and backward digit-span tasks were included to investigate transfer effects between the trained tasks and nontrained tasks.

### Testing procedure

Testing and training were carried out using an online platform developed by the research team and Cauldron, a software company (http://www.cauldron.sc). Participants completed each of the three test sessions in groups; adolescents were tested in school and adults were tested in a university computer room (for average group sizes per age group, see Table S6 in the Supplemental Material). Participants used laptops, tablets, or desktop computers. Responses on all five tasks were made using a mouse, touchpad, or touchscreen. Before each task, an experimenter gave instructions, and participants completed practice trials until they correctly completed three trials on each of the five tasks. Participants were given visual feedback on their performance in the practice trials only. Task order was counterbalanced among training groups and across test sessions using a Latin-square design. Because of school scheduling constraints, Test Session 1 was split over 2 or 3 days for four groups (see Table S6 in the Supplemental Material). All other sessions were completed in one sitting. To check whether this influenced the main results, we reran the analysis and excluded data from individuals whose test sessions were split over multiple days (see Supplementary Analyses in the Supplemental Material).

### Training procedure

Participants were asked to complete 20 days of training in any Internet-enabled device other than a smartphone. The training platform did not allow more than one training session to be started each day. Each training session lasted a maximum of 12 min or a set number of trials (for specific values, see each task’s Training Protocol section), whichever was reached first. If a participant failed to respond for more than 5 min, the training session timed out and was not included in the total number of training sessions. Task difficulty was adaptive according to performance within training sessions, and participants received feedback on their performance.

The training was designed to be motivating: We provided positive feedback, such as flashing stars, after every correct response. Motivational phrases (e.g., “awesome!” or “three in a row!”) were shown as intermittent reinforcers (Ferster & Skinner, 1957). To incentivize training further, participants received virtual trophies. Before each training session, participants were asked to select a trophy chest (bronze, silver, or gold); after the session, they could open the chest to find a trophy that would be displayed in their online trophy cabinet. Participants were able to track the number of training sessions they had completed by viewing their trophy cabinet. Participants were reminded about training by automated daily e-mails and additional e-mail reminders sent by the research team, and teachers were asked to remind adolescent participants to train. Volunteers also received monetary rewards at Test Session 2 if they had completed at least 15 training days. Adolescents received a £10 Amazon voucher, and adults received £30 in cash; after Test Session 3, adults received a further £10 in cash and adolescents received a certificate of participation. The training was designed to resemble school-based learning: Testing was carried out in groups in the classroom, and the training program was comparable with homework in terms of duration and frequency.

### Numerosity discrimination

The numerosity-discrimination task was used to measure the ability to rapidly approximate and compare the number of items within two different sets of colored dots presented on a gray background. In this task, the total number of dots and dot proportions (i.e., the relative number of dots of each color) in each array could be modified to vary difficulty level, such that a higher number of dots and a higher dot proportion represented a more difficult trial (Halberda et al., 2012).

#### Testing protocol

The dot proportions used were .3, .4, .42, .45, .47, and .49; the last four proportions, which were more difficult, appeared twice as often as the first two, easier proportions. The testing started with four easy trials (i.e., dot proportion = .3), but the proportion used in all subsequent trials was randomized. Only trials with black and white dots were included in the testing. Individual dot positions for each array were selected pseudorandomly: Their position was restricted such that none of the dots overlapped or touched and each dot was within the borders of the stimulus display.

Each trial started with a fixation cross presented for 250 ms, followed by a dot array presented for 200 ms. Participants were asked to select the color of the more numerous dots. The two possible response options were displayed at the same time as the dot array and stayed on the screen until a response was given. The position (i.e., left or right) of the response buttons (i.e., “black” or “white”) on the screen was counterbalanced between participants. There was no time limit on the response in each trial. After participants provided a response, the next trial started immediately. The numerosity-discrimination task took 7 min to complete.

#### Training protocol

Each training session took 12 min or 64 trials to complete, whichever was reached first. All possible dot proportions were used. The first training session started with an initial dot proportion of .3. After each correct trial, difficulty increased one level (i.e., dot proportion came closer to .5); after each incorrect trial, it decreased two levels. The initial difficulty of each subsequent training session was two levels lower than the peak difficulty encountered in the previous training session. In training, randomly selected pairs of colored dot sets were used (black and white, blue and yellow, blue and orange, violet and yellow, and violet and orange).

### Relational reasoning

A modified version of Raven’s Progressive Matrices (Raven, 1960) was used to examine the ability to detect abstract relationships between groups of items. In this version of the relational-reasoning task, puzzles consisted of a 3 × 3 matrix; eight of the cells contained shapes, but there was no shape in the bottom right cell. To select the correct response option, the participant had to deduce the pattern of change within the matrix. The items in a matrix could vary by color, size, shape, and position across the matrix.

#### Testing protocol

Each trial started with a 500-ms fixation cross, followed by a 100-ms blank screen. In each trial, a puzzle was presented on the left side of the screen, and four possible response options were shown on the right side of the screen. Each puzzle was presented for 30 s. After 25 s, a clock appeared above the response options, indicating that 5 s remained until the next trial. The next trial started after participants responded or after 30 s had elapsed. The task took 8 min to complete. There were three test sessions; a different set of 80 puzzles using abstract shapes was created for each session. The order of the 80 puzzles within each set was the same for all participants, starting with five easy trials. The order of the three sets was counterbalanced across participants. If a participant completed all 80 puzzles within the 8-min time limit, the same set was presented again, but data from these additional puzzles were not included in the analysis.

#### Training protocol

Each training session took 12 min or 40 trials to complete, whichever was reached first. For each session, abstract and iconic puzzle shapes were selected. The first training session started with an easy puzzle. Training was adapted to performance such that the number of changing dimensions increased by one after each correct response and decreased by one after each incorrect response. The initial difficulty of each subsequent training session was two levels lower than that in the previous training session.

### Face perception

The face-perception task measured the ability to process featural and configural changes in faces (Cohen Kadosh, 2011). Participants were asked to decide whether two faces presented consecutively were the same or different. Faces were considered to be different when there were changes in any of the following face properties: gaze direction (left or right), expression (happy or sad), or identity (Person A or Person B). Participants were informed that faces should be classified as the same only if all three face properties were exactly the same.

#### Testing protocol

Photos of 26 faces (16 white, 10 Asian; 16 female, 10 male), were taken under standardized lighting conditions for the purpose of this experiment. Four color photos were obtained for each face: two with a happy expression (one with leftward gaze and one with rightward gaze) and two with a sad expression (one with leftward gaze and one with rightward gaze). Photos were scaled to a uniform size and cropped to exclude external features of the face (e.g., hair).

Each trial started with a fixation cross presented for 800 ms, followed by the first face for 500 ms, and then another fixation cross for 800 ms, and then the second face for 500 ms. In the response display, the two possible response options (“same” or “different”) were shown simultaneously with the presentation of the two faces. The next trial started immediately after participants responded. One test took 7.5 min to complete.

Each test session contained a different set of stimuli, and each set comprised 48 different trials in which the faces of White women were shown. The order of the three sets of stimuli was counterbalanced across participants. If participants finished the 48 trials within the 7.5-min time limit, the trials were presented again, but the data were not included in the analysis. On the first 2 trials, the images had a noise mask of 25%, and difficulty in the remaining trials was increased by adding noise masks of increasing strength (from 25% to 81% in steps of 8 percentage points).

#### Training protocol

Each training session lasted for 12 min or 48 trials, whichever was reached first. Twenty different sets of faces (five sets showed Asian women, five sets showed Asian men, five sets showed white women, and five sets showed white men) were generated for training. Training task difficulty was adapted to performance. In the first training session, a 25% noise mask was applied to the first images. After a correct trial, noise strength was increased by 8 percentage points. After an incorrect trial, noise strength was decreased by 16 percentage points or kept at 25%—the lowest level. Each subsequent training session started with an initial difficulty level that was 16 percentage points lower than the peak difficulty encountered in the previous training session.

### Face-memory testing protocol

An adaptation of the Cambridge Face Memory Test (Duchaine & Nakayama, 2006) was used to assess the ability to learn and recognize unknown faces using a three-alternative forced-choice (3-AFC) trial. Participants were asked to memorize six target faces and then locate one of the targets from a panel of three faces. The panel comprised the target face plus two distractor faces that had not been memorized. A set of 198 face stimuli matching the specifications of the original Cambridge Face Memory Test was created for the purpose of the experiment. Black and white photographs of 66 white males taken from three angles (front, left quarter profile, and right quarter profile) were obtained from the Facial Recognition Technology database (Phillips, Moon, Rizvi, & Rauss, 2000). Photos were cropped to exclude external features of the face (e.g., hair) using the GNU Image Manipulation Program (GIMP Team, 2013). The task consisted of three blocks. In the first block, a target face was shown at three different angles, for 3 s each, and this was followed by three 3-AFC trials. This procedure was repeated for five more target faces. In the second block, frontal views of the same six target faces were presented simultaneously for 20 s, and this was followed by eighteen 3-AFC trials. In the third block, frontal views of the same six target faces were presented simultaneously for 20 s, but a 50% Gaussian noise mask was added to the faces in the eighteen 3-AFC trials that followed.

There was no time limit on the response in any of the blocks. After participants responded, the next trial started immediately. The task took 9 min or 54 trials to complete, whichever came first. Three sets of stimuli were created, one for each of the three test sessions. The order of presentation of these sets was counterbalanced across participants. Each testing set contained 6 unique target faces and 6 unique distractor faces, as well as a set of 30 distractor faces that was used in all three test sessions. These common distractors were used to increase the difficulty of the task and prevent ceiling effects.

### Backward digit-span testing protocol

The backward digit-span task was used to measure verbal working memory. Participants were asked to remember a sequence of digits in a certain order and to recall them in the reverse order. Minimum sequence length was two digits, sequences neither started nor ended with a 0, and no digit appeared twice or more in a row. Each trial started with a 500-ms fixation cross, followed by a 250-ms blank display. Digits were presented at a rate of one per second with an interstimulus interval of 250 ms. At the end of each sequence, participants were presented with a number of dashes equal to the length of the digit sequence they had just seen and were asked to input the digit sequence in reverse order, using the on-screen keyboard. Participants were not permitted to correct a response after a digit had been entered. There was no time limit on the response. After the response was given, the next trial started immediately. The task took 6 min to complete. The sequence length started at five digits, and trial difficulty was adapted to performance such that after correct trials, the difficulty level increased by one level (i.e., the sequence length increased by one), and after incorrect trials, the difficulty level decreased by 1 level (i.e., the sequence length decreased by 1).

### Analysis

Generalized linear mixed-effects models (GLMM) implemented in the lme4 (Bates, Maechler, & Bolker, 2014) package for the R software environment (R Development Core Team, 2013) were used to investigate the degree to which participants improved their task performance after training and whether the effect of training differed between age groups or between training groups. Trials in any of the tasks with a response time under 250 ms were excluded from the analysis. For the numerosity-discrimination, relational-reasoning, face-perception, and face-memory tasks, the sums of correct and incorrect responses across trials were used as dependent variables. The models predicted each participant’s task accuracy on the basis of four independent variables: training group, age group, test session, and number of completed training sessions (to control for differences in motivation). The model included fixed main effects of all four variables and fixed interaction effects between test session, training group, and age group as well as an interaction between training group and number of days trained. Helmert coding was used for all categorical fixed effects. Training days were standardized to *z* scores. To account for individual differences, attrition, and the repeated measures for each participant, the model included a participant-specific random intercept (nested in school or university).

A linear mixed-effects model was used to investigate training effects on performance in the backward digit-span task. This model incorporated each participant’s maximal digit span as the dependent variable and the same random and fixed effects that were used in the GLMMs. The effects of the predictors on the dependent variables were investigated using an omnibus Type III Wald χ^2^ test. Planned comparisons were performed to inspect differences across test sessions, age groups, and training groups using the multcomp package (Hothorn, Bretz, & Westfall, 2008). For each of the five tasks, we inspected 26 comparisons of performance changes between Test Sessions 1 and 2 and between Test Sessions 1 and 3. To investigate general training effects, we analyzed changes in performance in the trained tasks between test sessions within training groups (2 tests) and compared these effects between training groups (4 tests). Age-dependent training effects were investigated by looking at changes in performance in each age group on their trained task (8 tests). Between-age-group comparisons of age-dependent training effects were made by looking at changes in accuracy between age groups on their trained task (12 tests). All reported results were Bonferroni-corrected for these 26 comparisons. For additional analysis, which investigated potential confounds, see Supplementary Analyses in the Supplemental Material.

## Results

### Overall training effects

**Fig. 2.**
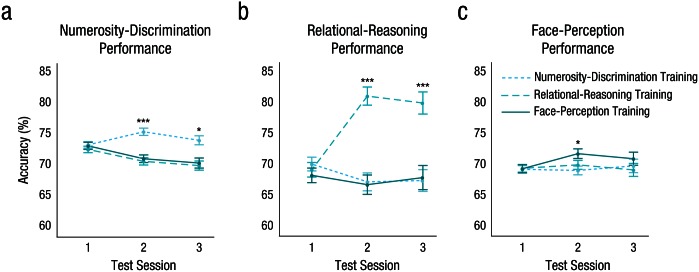
Accuracy results by training group. Percentage accuracy on the (a) numerosity-discrimination task, (b) relational-reasoning task, and (c) face-perception task is plotted as a function of test session. Asterisks in (a) and (b) indicate significantly greater gains at Test Sessions 2 and 3 in the group trained in the indicated task than in the other two groups (*p < .05, ***p < .001). The asterisk in (c) indicates a significant difference in gain at Test Session 2 between the group trained in face perception and the group trained on numerosity discrimination (*p < .05). Error bars indicate ±1 *SE*.

Training on the numerosity-discrimination, relational-reasoning, and face-perception tasks improved performance on these respective tasks (Fig. 2). Changes in performance differed between training groups, as indicated by significant interactions between test session and training group for the numerosity-discrimination task, χ^2^(4, *N* = 662) = 34.61, *p* < .001; relational-reasoning task, χ^2^(4, *N* = 661) = 328.48, *p* < .001; and face-perception task, χ^2^(4, *N* = 661) = 12.57, *p* = .014.

Planned comparisons showed that participants who were trained in numerosity discrimination showed significantly improved performance in numerosity discrimination at Test Session 2, *z* = 3.38, *p* < .02, but those gains were not sustained at Test Session 3, *z* = 1.39, n.s. Compared with participants who received training in one of the other two tasks, participants in the numerosity-discrimination training group showed significantly higher gains in numerosity discrimination at Test Session 2 (comparison with participants trained in relational reasoning: *z* = 5.24, *p* < .001; comparison with participants trained in face perception: *z* = 4.65, *p* < .001; see Table S1 in the Supplemental Material). These effects were due mainly to the adult age group. When the adults’ data were excluded, some of the effects of numerosity-discrimination training became nonsignificant after Bonferroni correction (see Table S7 in the Supplemental Material).

Participants who were trained in relational reasoning showed significantly improved performance in relational reasoning at Test Session 2, *z* = 16.52, *p* < .001, and Test Session 3, *z* = 12.1, *p* < .001. These gains were higher than those in participants trained in one of the other tasks (comparison with participants trained in numerosity discrimination; *z* = 15.55, *p* < .001; compared with participants trained in face perception; *z* = 14.89, *p* < .001; see Table S2 in the Supplemental Material).

Participants who were trained in face perception showed significantly improved performance in face perception at Test Session 2, *z* = 3.92, *p* < .003, but not at Test Session 3, *z* = 2.79, n.s. The gains at Test Session 2 were higher than those in participants trained in numerosity discrimination, *z* = 3.16, *p* < .05 (see Table S3 in the Supplemental Material). However, these effects were not stable in supplementary analyses: The training effects in face perception lost significance when we controlled for such confounding variables as variation in group size (see Tables S7–S9 in the Supplemental Material).

### Age-dependent training effects

**Fig. 3.**
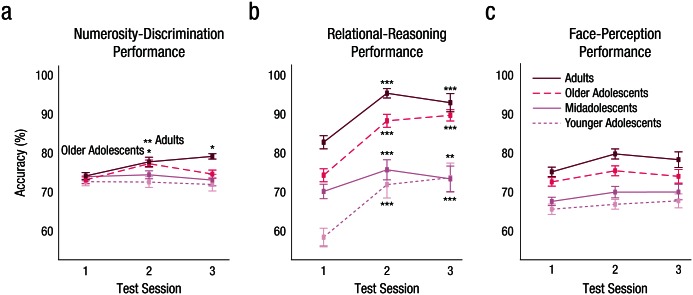
Accuracy results by age group. Percentage accuracy on the (a) numerosity-discrimination task, (b) relational-reasoning task, and (c) face-perception task is plotted as a function of test session. Asterisks indicate significant training gains at Test Session 2 or 3 within age groups (*p < .05, **p < .005, ***p < .001). Error bars indicate ±1 *SE*.

Age group significantly moderated the general training effects for the numerosity-discrimination task, χ^2^(12, *N* = 662) = 24.64, *p* < .02, and the relational-reasoning task, χ^2^(12, *N* = 661) = 80.14, *p* < .001, but not for the face-perception task, χ^2^(12, *N* = 661) = 8.80, n.s. (Fig. 3).

The only age groups to improve their performance in numerosity discrimination at Test Session 2 were older adolescents, *z* = 3.49, *p* < .02, and adults, *z* = 3.80, *p* < .005, who were trained in numerosity discrimination. These improvements were larger than the changes in performance in younger adolescents (comparison with older adolescents: *z* = −3.20, *p* < .05; compared with adults: *z* = −3.34, *p* < .05). Only adults showed a consolidation effect in numerosity discrimination at Test Session 3, *z* = 3.52, *p* < .02, and this effect was larger than that for midadolescents, *z* = −3.30, *p* < .05 (see Fig. S1 and Table S1 in the Supplemental Material). However, the training effects of numerosity discrimination did not remain statistically significant when we included covariates for differences in spacing of testing and group size at testing. This was particularly the case for the sustained training effects at Test Session 3 (see Tables S7–S9 in the Supplemental Material).

All age groups trained in relational reasoning showed improved performance in relational reasoning at Test Session 2 (younger adolescents: *z* = 6.11, *p* < .001; midadolescents: *z* = 5.17, *p* < .001; older adolescents: *z* = 11.53, *p* < .001; adults: *z* = 9.76, *p* < .001). Improvements were stronger in older adolescents and adults than in younger adolescents and midadolescents (younger adolescents vs. midadolescents: *z* = 0.97, n.s.; midadolescents vs. older adolescents: *z* = −5.87, *p* < .001; older adolescents vs. adults: *z* = −1.75, n.s.; see Fig. S1 and Table S2 in the Supplemental Material). Improvements were sustained at Test Session 3 in all age groups (younger adolescents: *z* = 5.57, *p* < .001; midadolescents: *z* = 3.68, *p* < .01; older adolescents: *z* = 9.36, *p* < .001; adults: *z* = 5.54, *p* < .001), but were stronger in older adolescents and adults than the younger age groups (younger adolescents vs. midadolescents: *z* = 1.63, n.s.; midadolescents vs. older adolescents: *z* = −5.74, *p* < .001; older adolescents vs. adults: *z* = 1.49, n.s.; see Fig. S1 and Table S2 in the Supplemental Material).

None of the contrasts for face perception training was significant (see Table S3 in the Supplemental Material).

### Transfer effects

There was no evidence of transfer from training in relational reasoning to backward digit span or from face perception to face memory.

The two-way interaction between test session and training group was not significant for performance on the backward digit-span task, χ^2^(4, *N* = 663) = 2.54, n.s., and no improvements at Test Session 2 or 3 were found in the relational-reasoning group (see Table S4 in the Supplemental Material). There was no effect of age group on transfer to the backward digit-span task, χ^2^(12, *N* = 663) = 14.87, n.s., and none of the age groups trained in relational reasoning improved their performance significantly in the backward digit-span task (see Table S4 in the Supplemental Material).

For the face-memory task, the two-way interaction between test session and training group was significant, χ^2^(4, *N* = 662) = 12.31, *p* < .02. However, no improvements in performance in the face-perception training group were found at Test Session 2 or 3 (see Table S5 in the Supplemental Material). There was no effect of age group on transfer to face memory, χ^2^(12, *N* = 662) = 13.31, n.s., and none of the age groups trained in face perception showed significantly improved performance in face memory (see Table S5 in the Supplemental Material).

## Discussion

This training study aimed to investigate cognitive skills relevant to math education and when during adolescence such skills are best trained. Although numerosity-discrimination training yielded small improvements only in late adolescence and adulthood, relational-reasoning training was already effective in early adolescence but showed a linear increase in benefit from mid- to late adolescence, and then no further improvement into adulthood. Training on face perception did not result in different levels of improvement in the different age groups. The results suggest that the ability to learn how to improve skill in numerosity discrimination and especially relational reasoning is greater in late than in early adolescence.

Overall, participants who were trained in numerosity discrimination improved their numerosity discrimination skills more than participants trained in the other tasks improved their skills in numerosity discrimination. However, these effects were age-dependent: Only older adolescents’ and adults’ performance improved significantly after training. Previous studies have shown that numerosity discrimination, which is related to mathematics performance, peaks at around the age of 30 (Halberda et al., 2012), and that approximate number processing can be trained in adulthood (DeWind & Brannon, 2012; Park & Brannon, 2013). However, ours is the first study to compare the training effect between age groups.

Relational-reasoning performance was more improved by relational-reasoning training than by training in other tasks. This training effect was observed in all age groups: Relational-reasoning training improved relational-reasoning task performance throughout adolescence and adulthood. This finding supports earlier results from previous research in which single age groups were studied (Mackey et al., 2011; Mackey, Whitaker, & Bunge, 2012). The effects survived a 6-month no-training period. Between-age-group comparisons showed that the benefit from relational-reasoning training increased from mid- to late adolescence, after which no further benefit was found in adulthood. We found a pattern of significantly higher improvement in relational reasoning in older adolescents and adults compared with younger age groups that was similar to the pattern observed in the numerosity-discrimination task. This finding provides further evidence that training during older adolescence results in greater improvements in performance than does training during early adolescence.

The fact that relational reasoning can be trained in all the age groups tested here, and that it is particularly amenable to training during late adolescence, does not support the notion that matrix reasoning gives an indication of some kind of innate, fixed ability. This has implications for education because matrix reasoning is commonly used in IQ tests and school entrance exams.

Participants who were trained in face perception showed improvements in identifying changes in faces and facial features compared with participants trained only in numerosity discrimination. There were no age-dependent training effects. Previous studies on face-perception training in adults have also yielded inconsistent results. For example, face-cognition speed training was found to be effective in adults, whereas face-memory training was not (Dolzycka et al., 2014).

There was no evidence of transfer between dissimilar skills from relational-reasoning training to working memory performance or from face-perception training to face-memory performance. A small number of studies have demonstrated transfer effects from a trained task to a nontrained task, particularly if they are closely related (Klingberg, 2010; Thorell et al., 2009). Many other studies have not (Owen et al., 2010). Future studies should investigate transfer to similar and dissimilar skills in a broader range of tasks and in a large age range to evaluate the significance of age-dependent transfer effects of education.

The increased effects of training during late adolescence and adulthood observed in the current study for numerosity discrimination and relational reasoning have several possible mechanistic explanations. First, improvements in training with age might be related to neurocognitive development. The prefrontal cortex develops particularly late (Tamnes et al., 2010) and may retain high levels of plasticity (Fuhrmann, Knoll, & Blakemore, 2015). Tasks that rely heavily on this region (Dumontheil, 2014), such as relational reasoning, may therefore be best trained late in development. Performance on executive-function tasks gradually improves throughout adolescence (Dumontheil et al., 2010), which might also contribute to improved learning with age. Until recently, most studies investigating neuroplasticity have concentrated on early childhood and have suggested that the adaptive processes of the nervous system are heightened in early development (Kuhl, 2004; Lewis & Maurer, 2005). In contrast, studies focusing on sensitive periods in later development are rare. Our findings indicate that the acquisition of relational reasoning and numerosity discrimination is more efficient in late adolescence than earlier in the teenage years, which suggests that plasticity for certain cognitive skills is sustained or even heightened at this relatively late stage of development. However, our study did not include participants younger than 11 years old, and we therefore cannot exclude the possibility that training would be efficient in younger participants. Future studies will need to elucidate the neurocognitive mechanisms of cognitive training and include younger as well as older age groups to show the trajectory of plasticity before and after adolescence.

Second, improved learning in late adolescence might be due to better use of strategy. Older adolescents and adults have greater general cognitive abilities than young and midadolescents (Gur et al., 2012), which might enable them to develop and deploy strategies that result in greater training improvements. Of the three trained tasks, relational reasoning might be most amenable to improvement through enhanced cognitive strategies (Goodwin & Johnson-Laird, 2005).

Third, the age-dependent training effects might be due to a number of confounding variables. The testing and training conditions and behavior were similar for the three adolescent groups, but the adult group was unavoidably different from the adolescent groups, in that the adults were self-selected and were paid more for taking part than were the adolescents. The adolescent groups were self-selected to a lesser degree in that entire school classes took part. Given these differences, as might be expected, adults trained more and completed their training more quickly than adolescents. There were also differences between age groups in the spacing between test sessions and group sizes at testing. We controlled for these possible confounds by including the number of training days as a covariate in our main statistical analyses. In addition, supplementary analysis showed that excluding the adult data, or including covariates for confounds named above, resulted in no qualitative differences: The interactions remained significant and effects were still in the same direction. However, although the training and age effects of relational reasoning were remarkably robust, the effects of numerosity discrimination were weaker and should therefore be interpreted with caution.

## Conclusion

We found that complex cognitive skills relevant to mathematics education, particularly relational reasoning, show larger training effects in late adolescence than earlier in adolescence. These findings highlight the importance of late adolescence for education and, in contrast to the common assumption that earlier learning is better, highlight the need to investigate late adolescence as a potential window of opportunity for educational interventions.

## Supplementary Material

Supplementary material
