# Supplementary material for: A Window of Opportunity for Cognitive Training in Adolescence
Source: Psychol Sci. 2016 Nov 4;27(12):1620–31. doi: 10.1177/0956797616671327 (PMC5221734; doi:10.1177/0956797616671327)
Supplement: Supplementary material [file DS_10.1177_0956797616671327.pdf]

DOI: 10.1177/0956797616671327

Supplemental Material

A window of opportunity for cognitive training in adolescence

Lisa. J. Knoll*[1#], Delia Fuhrmann*[1], Ashok L. Sakhardande[1], Fabian Stamp[1], Maarten Speekenbrink[2], Sarah-Jayne Blakemore[1]

* shared first author

# corresponding author

Affiliations:

[1] Institute of Cognitive Neuroscience, University College London, London, United Kingdom

[2] Department of Experimental Psychology, University College London, London, United Kingdom

Running head: cognitive training in adolescence

DOI: 10.1177/0956797616671327

Results

General training effects

Table S1. Planned contrasts of numerosity discrimination performance change between test sessions.

	**Estimate**	**SE**	**z-ratio**	**p**	**p (bonf.)**	
TS1 vs. TS2, ND	0.39	0.11	3.38	< .001	.019	*
TS1 vs. TS3, ND	0.18	0.13	1.39	.164	1	
TS1 vs. TS2, ND vs. FP	0.75	0.16	4.65	< .001	< .001	***
TS1 vs. TS2, ND vs. RR	0.85	0.16	5.24	< .001	< .001	***
TS1 vs. TS3, ND vs. FP	0.58	0.18	3.15	.002	.042	*
TS1 vs. TS3, ND vs. RR	0.63	0.18	3.50	< .001	.012	*
TS1 vs. TS2, ND, younger adol.	-0.07	0.06	-1.11	.268	1	
TS1 vs. TS2, ND, mid-adol.	0.06	0.06	0.95	.344	1	
TS1 vs. TS2, ND, older adol.	0.20	0.06	3.49	< .001	.013	*
TS1 vs. TS2, ND, adults	0.20	0.05	3.80	< .001	.004	**
TS1 vs. TS3, ND, younger adol.	-0.05	0.07	-0.74	.460	1	
TS1 vs. TS3, ND, mid-adol.	-0.07	0.07	-1.08	.278	1	
TS1 vs. TS3, ND, older adol.	0.06	0.06	0.99	.324	1	
TS1 vs. TS3, ND, adults	0.24	0.07	3.52	< .001	.011	*
TS1 vs. TS2, ND, younger adol. vs. mid-adol.	-0.12	0.08	-1.45	.146	1	
TS1 vs. TS2, ND, mid-adol. vs. older adol.	-0.14	0.08	-1.74	.082	1	
TS1 vs. TS2, ND, older adol. vs. adults	0.00	0.08	-0.06	.953	1	
TS1 vs. TS2, ND, younger adol. vs. adults	-0.27	0.08	-3.34	< .001	.022	*
TS1 vs. TS2, ND, mid-adol. vs. adults	-0.15	0.08	-1.85	.065	1	
TS1 vs. TS2, ND, younger adol. vs. older adol.	-0.26	0.08	-3.20	.001	.036	*
TS1 vs. TS3, ND, younger adol. vs. mid-adol.	0.02	0.09	0.24	.811	1	
TS1 vs. TS3, ND, mid-adol. vs. older adol.	-0.13	0.09	-1.47	.142	1	
TS1 vs. TS3, ND, older adol. vs. adults	-0.18	0.09	-2.03	.043	1	
TS1 vs. TS3, ND, younger adol. vs. adults	-0.29	0.10	-3.06	.002	.059	
TS1 vs. TS3, ND, mid-adol. vs. adults	-0.31	0.09	-3.30	< .001	.025	*
TS1 vs. TS3, ND, younger adol. vs. older adol.	-0.11	0.09	-1.21	.225	1	

Abbreviations: TS1 = Test Session 1; TS2 = Test Session 2; TS3 = Test Session 3; ND = numerosity discrimination training, RR = relational reasoning training, FP = face perception training, younger adol. = younger adolescents, mid-adol. = mid-adolescents, older adol. = older adolescents, * $p < 0.05$, ** $p < 0.01$,*** $p < .001$

DOI: 10.1177/0956797616671327

Table S2. Planned contrasts of relational reasoning performance change between test sessions.

	**Estimate**	**SE**	**z-ratio**	**p**	**p (bonf.)**	
TS1 vs. TS2, RR	3.17	0.19	16.52	<.001	<.001	***
TS1 vs. TS3, RR	2.59	0.21	12.1	<.001	<.001	***
TS1 vs. TS2, RR vs. FP	3.76	0.25	14.89	<.001	<.001	***
TS1 vs. TS2, RR vs. ND	3.90	0.25	15.55	<.001	<.001	***
TS1 vs. TS3, RR vs. FP	3.11	0.29	10.8	<.001	<.001	***
TS1 vs. TS3, RR vs. ND	3.29	0.29	11.33	<.001	<.001	***
TS1 vs. TS2, RR, younger adol.	0.46	0.08	6.11	<.001	<.001	***
TS1 vs. TS2, RR, mid-adol.	0.36	0.07	5.17	<.001	<.001	***
TS1 vs. TS2, RR, older adol.	1.03	0.09	11.53	<.001	<.001	***
TS1 vs. TS2, RR, adults	1.32	0.13	9.76	<.001	<.001	***
TS1 vs. TS3, RR, younger adol.	0.46	0.08	5.57	<.001	<.001	***
TS1 vs. TS3, RR, mid-adol.	0.28	0.08	3.68	<.001	.006	**
TS1 vs. TS3, RR, older adol.	1.06	0.11	9.36	<.001	<.001	***
TS1 vs. TS3, RR, adults	0.79	0.14	5.54	<.001	<.001	***
TS1 vs. TS2, RR, younger adol. vs. mid-adol.	0.10	0.10	0.97	.330	1	
TS1 vs. TS2, RR, mid-adol. vs. older adol.	-0.67	0.11	-5.87	<.001	<.001	***
TS1 vs. TS2, RR, older adol. vs. adults	-0.28	0.16	-1.75	.080	1	
TS1 vs. TS2, RR, younger adol. vs. adults	-0.85	0.15	-5.51	<.001	<.001	***
TS1 vs. TS2, RR, mid-adol. vs. adults	-0.95	0.15	-6.27	<.001	<.001	***
TS1 vs. TS2, RR, younger adol. vs. older adol.	-0.57	0.12	-4.84	<.001	<.001	***
TS1 vs. TS3, RR, younger adol. vs. mid-adol.	0.18	0.11	1.63	.104	1	
TS1 vs. TS3, RR, mid-adol. vs. older adol.	-0.78	0.14	-5.74	<.001	<.001	***
TS1 vs. TS3, RR, older adol. vs. adults	0.27	0.18	1.49	.136	1	
TS1 vs. TS3, RR, younger adol. vs. adults	-0.33	0.16	-2.00	.046	1	
TS1 vs. TS3, RR, mid-adol. vs. adults	-0.51	0.16	-3.17	.002	.040	*
TS1 vs. TS3, RR, younger adol. vs. older adol.	-0.60	0.14	-4.28	<.001	<.001	***

Abbreviations: TS1 = Test Session 1; TS2 = Test Session 2; TS3 = Test Session 3; ND = numerosity discrimination training; RR = relational reasoning training; FP =face perception training; younger adol. = younger adolescents; mid-adol. = mid-adolescents; older adol. = older adolescents. * $p < 0.05$, ** $p < 0.01$,*** $p < .001$

DOI: 10.1177/0956797616671327

Table S3. Planned contrasts of face perception performance change between test sessions.

	Estimate	SE	z-ratio	p	p (bonf.)	
TS1 vs. TS2, FP	0.54	0.14	3.92	<.001	.002	**
TS1 vs. TS3, FP	0.46	0.16	2.79	.005	.137	
TS1 vs. TS2, FP vs. RR	0.46	0.19	2.43	.015	.393	
TS1 vs. TS2, FP vs. ND	0.59	0.19	3.16	.002	.042	*
TS1 vs. TS3, FP vs. RR	0.48	0.22	2.12	.034	.882	
TS1 vs. TS3, FP vs. ND	0.34	0.23	1.50	.133	1	
TS1 vs. TS2, FP, younger adol.	0.04	0.06	0.65	.515	1	
TS1 vs. TS2, FP, mid-adol.	0.12	0.06	2.08	.038	.985	
TS1 vs. TS2, FP, older adol.	0.13	0.07	1.91	.056	1	
TS1 vs. TS2, FP, adults	0.25	0.09	2.89	.004	.100	
TS1 vs. TS3, FP, younger adol.	0.09	0.06	1.39	.164	1	
TS1 vs. TS3, FP, mid-adol.	0.13	0.06	1.99	.046	1	
TS1 vs. TS3, FP, older adol.	0.08	0.09	0.85	.393	1	
TS1 vs. TS3, FP, adults	0.17	0.10	1.59	.111	1	
TS1 vs. TS2, FP, younger adol. vs. mid-adol.	-0.08	0.08	-1.01	.314	1	
TS1 vs. TS2, FP, mid-adol. vs. older adol.	-0.01	0.09	-0.13	.899	1	
TS1 vs. TS2, FP, older adol. vs. adults	-0.12	0.11	-1.06	.290	1	
TS1 vs. TS2, FP, younger adol. vs. adults	-0.21	0.10	-2.03	.042	1	
TS1 vs. TS2, FP, mid-adol. vs. adults	-0.13	0.10	-1.24	.217	1	
TS1 vs. TS2, FP, younger adol. vs. older adol.	-0.09	0.09	-1.04	.297	1	
TS1 vs. TS3, FP, younger adol. vs. mid-adol.	-0.04	0.09	-0.43	.666	1	
TS1 vs. TS3, FP, mid-adol. vs. older adol.	0.05	0.11	0.48	.632	1	
TS1 vs. TS3, FP, older adol. vs. adults	-0.09	0.14	-0.66	.511	1	
TS1 vs. TS3, FP, younger adol. vs. adults	-0.08	0.12	-0.63	.531	1	
TS1 vs. TS3, FP, mid-adol. vs. adults	-0.04	0.12	-0.31	.760	1	
TS1 vs. TS3, FP, younger adol. vs. older adol.	0.01	0.11	0.12	.903	1	

Abbreviations: TS1 = Test Session 1; TS2 = Test Session 2; TS3 = Test Session 3; ND = numerosity discrimination training; RR = relational reasoning training; FP = face perception training; younger adol. = younger adolescents; mid-adol. = mid-adolescents; older adol. = older adolescents. * $p < 0.05$, ** $p < 0.01$,*** $p < .001$

DOI: 10.1177/0956797616671327

Age-dependent training effects

Table S4. Planned contrasts of digit span performance change between test sessions.

	Estimate	SE	z-ratio	p	p (bonf.)
TS1 vs. TS2, RR	0.52	0.32	1.61	.107	1
TS1 vs. TS3, RR	0.84	0.38	2.23	.026	.671
TS1 vs. TS2, RR vs. FP	-0.56	0.46	-1.21	.225	1
TS1 vs. TS2, RR vs. ND	-0.14	0.45	-0.30	.765	1
TS1 vs. TS3, RR vs. FP	0.19	0.55	0.35	.723	1
TS1 vs. TS3, RR vs. ND	0.04	0.54	0.07	.948	1
TS1 vs. TS2, RR, younger adol.	0.10	0.16	0.63	.529	1
TS1 vs. TS2, RR, mid-adol.	0.13	0.14	0.92	.356	1
TS1 vs. TS2, RR, older adol.	0.45	0.16	2.91	.004	.094
TS1 vs. TS2, RR, adults	-0.16	0.19	-0.85	.396	1
TS1 vs. TS3, RR, younger adol.	0.18	0.18	0.99	.324	1
TS1 vs. TS3, RR, mid-adol.	-0.08	0.16	-0.46	.645	1
TS1 vs. TS3, RR, older adol.	0.48	0.18	2.65	.008	.207
TS1 vs. TS3, RR, adults	0.25	0.22	1.14	.254	1
TS1 vs. TS2, RR, younger adol. vs. mid-adol.	-0.03	0.21	-0.14	.887	1
TS1 vs. TS2, RR, mid-adol. vs. older adol.	-0.32	0.21	-1.54	.125	1
TS1 vs. TS2, RR, older adol. vs. adults	0.61	0.25	2.51	.012	.318
TS1 vs. TS2, RR, younger adol. vs. adults	0.26	0.25	1.06	.291	1
TS1 vs. TS2, RR, mid-adol. vs. adults	0.29	0.24	1.23	.218	1
TS1 vs. TS2, RR, younger adol. vs. older adol.	-0.35	0.22	-1.59	.112	1
TS1 vs. TS3, RR, younger adol. vs. mid-adol.	0.26	0.25	1.04	.297	1
TS1 vs. TS3, RR, mid-adol. vs. older adol.	-0.56	0.25	-2.29	.022	.578
TS1 vs. TS3, RR, older adol. vs. adults	0.23	0.29	0.81	.420	1
TS1 vs. TS3, RR, younger adol. vs. adults	-0.07	0.29	-0.25	.801	1
TS1 vs. TS3, RR, mid-adol. vs. adults	-0.33	0.28	-1.19	.233	1
TS1 vs. TS3, RR, younger adol. vs. older adol.	-0.30	0.26	-1.18	.238	1

Abbreviations: TS1 = Test Session 1; TS2 = Test Session 2; TS3 = Test Session 3; ND = numerosity discrimination training; RR = relational reasoning training; FP = face perception training; younger adol. = younger adolescents; mid-adol. = mid-adolescents; older adol. = older adolescents. * $p < 0.05$, ** $p < 0.01$,*** $p < .001$

DOI: 10.1177/0956797616671327

Table S5. Planned contrasts of Cambridge Face Memory Task performance change between test sessions.

	Estimate	SE	z-ratio	p	p (bonf.)	
TS1 vs. TS2, FP	-0.59	0.14	-4.20	<.001	<.001	***
TS1 vs. TS3, FP	-0.78	0.17	-4.53	<.001	<.001	***
TS1 vs. TS2, FP vs. RR	0.29	0.20	1.48	.139	1	
TS1 vs. TS2, FP vs. ND	0.50	0.19	2.57	.010	.262	
TS1 vs. TS3, FP vs. RR	-0.31	0.24	-1.31	.189	1	
TS1 vs. TS3, FP vs. ND	-0.17	0.24	-0.71	.480	1	
TS1 vs. TS2, FP, younger adol.	-0.29	0.06	-4.84	<.001	<.001	***
TS1 vs. TS2, FP, mid-adol.	-0.21	0.06	-3.56	<.001	.010	**
TS1 vs. TS2, FP, older adol.	0.01	0.07	0.08	.936	1	
TS1 vs. TS2, FP, adults	-0.11	0.09	-1.22	.222	1	
TS1 vs. TS3, FP, younger adol.	-0.16	0.07	-2.25	.025	.640	
TS1 vs. TS3, FP, mid-adol.	-0.29	0.06	-4.52	<.001	<.001	***
TS1 vs. TS3, FP, older adol.	-0.14	0.09	-1.52	.129	1	
TS1 vs. TS3, FP, adults	-0.19	0.11	-1.74	.083	1	
TS1 vs. TS2, FP, younger adol. vs. mid-adol.	-0.08	0.08	-0.94	.346	1	
TS1 vs. TS2, FP, mid-adol. vs. older adol.	-0.21	0.09	-2.27	.023	.607	
TS1 vs. TS2, FP, older adol. vs. adults	0.11	0.11	0.99	.325	1	
TS1 vs. TS2, FP, younger adol. vs. adults	-0.18	0.11	-1.70	.089	1	
TS1 vs. TS2, FP, mid-adol. vs. adults	-0.10	0.11	-0.96	.337	1	
TS1 vs. TS2, FP, younger adol. vs. older adol.	-0.29	0.09	-3.09	.002	.053	
TS1 vs. TS3, FP, younger adol. vs. mid-adol.	0.13	0.10	1.33	.185	1	
TS1 vs. TS3, FP, mid-adol. vs. older adol.	-0.15	0.11	-1.34	.180	1	
TS1 vs. TS3, FP, older adol. vs. adults	0.05	0.14	0.34	.732	1	
TS1 vs. TS3, FP, younger adol. vs. adults	0.03	0.13	0.20	.840	1	
TS1 vs. TS3, FP, mid-adol. vs. adults	-0.10	0.13	-0.81	.420	1	
TS1 vs. TS3, FP, younger adol. vs. older adol.	-0.02	0.12	-0.19	.848	1	

Abbreviations: TS1 = Test Session 1; TS2 = Test Session 2; TS3 = Test Session 3; ND = numerosity discrimination training; RR = relational reasoning training; FP = face perception training; younger adol. = younger adolescents; mid-adol. = mid-adolescents; older adol. = older adolescents. * $p < 0.05$, ** $p < 0.01$,*** $p < .001$

DOI: 10.1177/0956797616671327

Supplementary Analyses

Confounding variables

We tested whether participants varied by age group and training group in a number of potential confounds (Table S6).

Table S6. Characteristics of the different age groups: number of days trained, days between training sessions, days between Test Session 1 (TS1) and Test Session 2 (TS2), days between TS2 and Test Session 3 (TS3), group size at testing, number of test sessions split over several days, number of participants no present at TS2 and number of participants not present at TS3.

	**Younger adolescents 11.27-13.38**	**Mid-adolescents 13.39-15.89**	**Older adolescents 15.90-18.00**	**Adults 18.01-33.15**	Test of difference
Number of days trained (Mean/SE)	14.90 (0.48)	14.74 (0.44)	14.62 (0.45)	18.83 (0.54)	$F(3,535)=15.53$, $p < .001$[1]
Days between training sessions (Mean/SE)	2.05 (0.15)	2.06 (0.14)	2.53 (0.15)	1.48 (0.17)	$F(3,535)=7.25$, $p < .001$[2]
Days between TS1 and TS2 (Mean/SE)	36.73 (0.69)	32.91 (0.67)	39.71 (0.73)	31.95 (0.89)	$F(3,596)=22.36$, $p < .001$[3]
Days between TS2 and TS3 (Mean/SE)	134.10 (2.71)	145.96 (2.58)	123.97 (2.96)	150.14 (3.84)	$F(3,390)=14.64$, $p < .001$[4]
Group size at testing (Mean/SE)	14.36 (1.98)	14.93 (1.95)	23.51 (2.00)	7.09 (8.00)	($\chi2(3) = 134.38$, $p < .001$)[5]
Number of test sessions split over 2 or 3 days	34	4	0	0	($\chi2(3) = 72.02$, $p < .001$)[6]
Number of participants not present at TS2	15	9	34	3	($\chi2(3) = 27.93$, $p < .001$)[7]
Number of participants not present at TS3	68	56	86	48	($\chi2(3) = 12.74$, $p = .005$)[8]

[1] Adults trained more than all other age groups ($p < .001$).
[2] Adults' training sessions were spaced more closely than older adolescents' ($p < .001$).
[3] Only adults' and mid-adolescents' TS1 and TS2 were similarly spaced, all other comparisons differed at $p < 0.05$.
[4] TS2 and TS3 of adults and mid-adolescents, and older and younger adolescents, were similarly spaced; all other comparisons differed at $p < 0.05$.
[5] Group size at test sessions differed between older and younger adolescents, and between older and mid-adolescents, at $p < .001$.
[6] There were more younger adolescents with split test sessions than all other age groups ($p < .001$); all other comparisons were non-significant.
[7] There were more older adolescents missing at TS2 than all other age groups ($p < 0.05$); all other comparisons were non-significant.

[8] There were more older adolescents missing at TS2 than mid-adolescents (*p* = .012); all other comparisons were non-significant.

There were no differences between training groups but age groups varied in the number of days they trained, the spacing between training and test sessions, number of participants missing at TS2 and TS3 and their group size at testing. Missing data was addressed by our main analyses (see Methods section). Because of the other differences between age groups, we tested whether the following potential confounds could explain our main findings:

<u>Differences between adults and the other age groups in the amount of training completed and the spacing between test sessions</u>

Adults completed more training than all other age groups and completed training more quickly than older adolescents (Table S6). To check whether this affected our main findings, we re-ran the models for the three training tasks (numerosity discrimination, relational reasoning, face perception) and excluded adults' data.

Again, the results were qualitatively similar, but there were some quantitative changes. One interaction and five planned contrasts became non-significant (Table S7):

- *Numerosity discrimination:* Some of the training effects for numerosity discrimination training became non-significant, particularly at TS3.
- *Relational reasoning:* No changes.
- *Face perception:* The interaction between time point and training group became non-significant for the face perception task in this analysis ($\chi2(4) = 8.52$, $p = .74$, previously $p = .014$). The training effects in face perception lost significance.

All other reported effects remained the same.

Table S7. Planned contrasts that became non-significant after excluding adults' data.

Original analysis					
**Contrast**	**Estimate**	**SE**	**z-ratio**	**p**	**p (bonf.)**
TS1 vs. TS2, ND	0.39	0.11	3.38	< .001	.019
TS1 vs. TS3, ND vs. FP	0.58	0.18	3.15	.002	.042
TS1 vs. TS3, ND vs. RR	0.63	0.18	3.50	< .001	.012
TS1 vs. TS2, FP	0.54	0.14	3.92	<.001	.002
TS1 vs. TS2, FP vs. ND	0.59	0.19	3.16	.002	.042
**New analysis without adults' data**					
**Contrast**	**Estimate**	**SE**	**z-ratio**	**p**	**p (bonf.)**
TS1 vs. TS2, ND	0.19	0.1	1.83	.067	1
TS1 vs. TS3, ND vs. FP	0.30	0.16	1.90	.057	1
TS1 vs. TS3, ND vs. RR	0.30	0.16	1.94	.053	.950
TS1 vs. TS2, FP	0.29	0.11	2.71	.007	.123
TS1 vs. TS2, FP vs. ND	0.36	0.15	2.46	.014	.253

Abbreviations: TS1 = Test Session 1; TS2 = Test Session 2; TS3 = Test Session 3; ND = numerosity discrimination training; RR = relational reasoning training; FP = face perception training

<u>Age-group differences in the spacing between test sessions and group sizes at testing</u>

The spacing between test sessions as well as group sizes at testing differed because of schools' timetabling constraints (Table S6).

To check whether this influenced our main results, we re-ran the models for the three training tasks (numerosity discrimination, relational reasoning, face perception) and included covariates for spacing between test sessions and group sizes.

Our results were qualitatively similar: the overall interactions were still significant and the effects went in the same direction. However, there were some quantitative changes in that nine planned contrasts that were significant in our main analysis now became non-significant

after Bonferroni-correction (three without Bonferroni-correction; Table S8). This may partly be due to the loss of power incurred by including additional covariates.

- *Numerosity discrimination:* Some of the training effects for numerosity discrimination training became non-significant, particularly at TS3.
- *Relational reasoning:* Younger and mid-adolescents did not show a training effect.
- *Face perception:* The overall training effect disappeared.

All other reported effects remained the same.

Table S8. Planned contrasts that became non-significant after including covariates for spacing between test sessions and group sizes

**Original analysis**					
**Contrast**	**Estimate**	**SE**	**z-ratio**	**p**	**p (bonf.)**
TS1 vs. TS2, ND	0.39	0.11	3.38	< .001	.019
TS1 vs. TS3, ND vs. FP	0.58	0.18	3.15	.002	.042
TS1 vs. TS2, ND, older adol.	0.20	0.06	3.49	< .001	.013
TS1 vs. TS2, ND, adults	0.20	0.05	3.80	< .001	.004
TS1 vs. TS3, ND, adults	0.24	0.07	3.52	< .001	.011
TS1 vs. TS3, RR, younger adol.	0.46	0.08	5.57	<.001	<.001
TS1 vs. TS3, RR, mid-adol.	0.28	0.08	3.68	<.001	.006
TS1 vs. TS2, FP	0.54	0.14	3.92	<.001	.002
**New analysis with covariates**					
**Contrast**	**Estimate**	**SE**	**z-ratio**	**p**	**p (bonf.)**
TS1 vs. TS2, ND	0.26	0.14	1.86	.063	1
TS1 vs. TS3, ND vs. FP	0.54	0.18	2.93	.003	.091
TS1 vs. TS2, ND, older adol.	0.16	0.06	2.6	.009	.246
TS1 vs. TS2, ND, adults	0.17	0.06	3.10	.002	.051
TS1 vs. TS3, ND, adults	0.08	0.12	0.63	.536	1
TS1 vs. TS3, RR, younger adol.	0.42	0.17	2.51	.012	.311
TS1 vs. TS3, RR, mid-adol.	0.24	0.17	1.43	.1752	1
TS1 vs. TS2, FP	0.35	0.17	2.07	.038	.996

Abbreviations: TS1 = Test Session 1; TS2 = Test Session 2; TS3 = Test Session 3; ND = numerosity discrimination training; RR = relational reasoning training; FP = face perception training; younger adol. = younger adolescents; mid-adol. = mid-adolescents; older adol. = older adolescents.

Testing group sessions were sometimes split

Testing was sometimes split over 2 or 3 sessions due to participants' time-constraints (Table S6).

To check whether this influenced the main results, we re-ran the models for the three training tasks (numerosity discrimination, relational reasoning, face perception) and excluded data from individuals whose test sessions were split over several days.

There were minor quantitative changes. Two planned contrasts became non-significant after Bonferroni-correction only (Table S9):

- *Numerosity discrimination:* Older adolescents' training effect was not significantly stronger than younger adolescents' training effects.
- *Relational reasoning:* The sustained training effect at TS3 was no longer significant.
- *Face perception:* No changes.

All other reported effects remained the same.

Table S9. Planned contrasts that became non-significant after excluding data from individuals whose test sessions were split over several days

**Original analysis**					
	**Estimate**	**SE**	**z-ratio**	**p**	**p (bonf.)**
TS1 vs. TS2, ND, younger adol. vs. older adol.	-0.26	0.08	-3.20	.001	.036
TS1 vs. TS3, RR, mid-adol.	0.28	0.08	3.68	<.001	.006
**New analysis without split training sessions**					
**contrast**	**Estimate**	**SE**	**z-ratio**	**p**	**p (bonf.)**
TS1 vs. TS2, ND, younger adol. vs. older adol.	-0.26	0.09	-2.99	.003	.073
TS1 vs. TS3, RR, mid-adol.	0.20	0.08	2.66	.008	.201

Abbreviations: TS1 = Test Session 1; TS2 = Test Session 2; TS3 = Test Session 3; ND = numerosity discrimination training; RR = relational reasoning training; FP = face perception training; younger adol. = younger adolescents; mid-adol. = mid-adolescents; older adol. = older adolescents.
